# MS Location Estimation Based on the Artificial Bee Colony Algorithm

**DOI:** 10.3390/s20195597

**Published:** 2020-09-29

**Authors:** Chien-Sheng Chen, Jen-Fa Huang, Nan-Chun Huang, Kai-Sheng Chen

**Affiliations:** 1 Department of Information Management, Tainan University of Technology, Tainan 701, Taiwan; t00243@mail.tut.edu.tw; 2 Department of Electrical Engineering, National Cheng Kung University, Tainan 710, Taiwan; huajf@ee.ncku.edu.tw (J.-F.H.); number17471747@gmail.com (N.-C.H.); 3 School of Electrical and Computer Engineering, Nanfang College of Sun Yat-Sen University, Guangzhou 510970, China

**Keywords:** time of arrival (TOA), non-line-of-sight (NLOS), artificial bee colony (ABC), mobile station (MS), base station (BS)

## Abstract

With the mature technology of wireless communications, the function of estimating the mobile station (MS) position has become essential. Suppressing the bias resulting from non-line-of-sight (NLSO) scenarios is the main issue for a wireless location network. The artificial bee colony (ABC) algorithm, based on the depiction of bee swarm’s foraging characteristics, is widely applied to solve optimization problems in several fields. Based on three measurements of time-of-arrival (TOA), an objective function is used to quantify the additional NLOS error on the MS positioning scheme. The ABC algorithm is adopted to locate the most precise MS location by minimizing the objective function value. The performance of the proposed positioning methods is verified under various error distributions through computer simulations. Meanwhile, the localization accuracy achieved by other existing methods is also investigated. According to the simulation results, accurate estimation of the MS position is derived and therefore the efficiency of the localization process is increased.

## 1. Introduction

With the rapid development of communication technology, the ability of searching for a mobile station (MS) location has become increasingly important in recent years. High-accuracy positioning can provide many commercial services for the users of wireless devices. Location-based services (LBSs) are used for car tracking, map navigation, and security applications. Among these, E-911, an emergency provision system regulated by the U.S. Federal Communications Commission (FCC), is one of the most well-known localization applications [1].

Generally, the propagation time can be measured by the MS and the base station (BS) in wireless cellular systems. Kinds of measurements are taken by the signals that travel between the MS and the BSs. According to different measurements, different known wireless location techniques are applied. Some of the most common schemes are the signal strength [2], angle of arrival (AOA) [3], time of arrival (TOA) [4], and time difference of arrival (TDOA) [5]. In order to provide better accuracy, hybrid TOA and AOA methods were proposed [6]. In [7], the authors utilized the improved linear-line-of-position to propose a TOA-based localization method for a 3-D environment. The accuracy of MS location estimation is affected by many factors, such as multipath, hearability, and non-line-of-sight (NLOS) propagation. One of the primary tasks for accurate location estimates in wireless communication location systems is NLOS propagation derived from the blocking of direct paths. NLOS often happens in an urban environment and can be considered as a dominant issue for time-based location estimation. The additional propagation time or distance may lead to significant degradations on location performance. In an ideal case, if the line-of-sight (LOS) propagation exists, the MS location can be derived successfully without error interference. However, an LOS propagation channel is hard to come by in a real environment.

To meet the high location accuracy, many location estimation algorithms were proposed to mitigate the NLOS error. To handle the problem of NLOS error, an omnidirectional mobile target-node (TN) localization technique with TOA and AOA measurements found by the antenna arrays was proposed, and the NLOS TN localization was theoretically derived based on the equations for NLOS identification [8]. In [9], the authors present an algorithm to classify the former into LOS and NLOS links by applying the propagation delay (PD) range estimations. A particle filter localization algorithm was proposed regarding the model for accounting NLOS conditions, and this method is feasible to simplify the working assumptions [10]. In [11], the authors proposed a non-iterative hybrid least square estimator to appraise the mobile unit (MU) coordinates using the TDOA measurements and direction of arrival estimates. For the reasons of NLOS interference reduction, the authors of this paper proposed several geometrical location methods by applying TOA measurements and AOA measurements simultaneously [12,13]. The authors put forward a method that combines the hybrid TOA/AOA measurements and short-range TOA measurements to appraise the location [14]. A model is proposed to simulate the situation that MS was in the NLOS transmission environment, whereas BSs have some links with the MS in an LOS environment.

In recent years, a lot of algorithms have been widely employed to resolve various constrained optimization problems. Optimization techniques can be categorized into two types, evolutionary algorithms (EAs) and swarm intelligence-based algorithms. The genetic algorithm, designated as following the evolution procedures in nature, is one of the most famous optimization methods among EAs. The whale optimization algorithm was proposed in [15], and its application on mobile positioning has been published. The authors in [16] used the distance measurements to derive the overlap of all the base stations and find out the unknown mobile station in the overlap by WOA. The artificial bee colony (ABC) algorithm adopts bee swarm activities and therefore it belongs to the category of swarm intelligence algorithms. Honey bees have several behaviors, such as the waggle dance, to search for better food sources in a natural environment. The ABC algorithm was proposed by Karaboga first in 2005 [17]. This algorithm provides a widely used optimal technology for solving multivariable function’s approach to various applications. For example, a better solution based on an ABC alternative was provided for vehicle routing problems [18]. Setting the parameters of the digital filter is important to the output signal, and the authors designed an infinite impulse response (IIR) to adjust the parameters by the ABC algorithm until the error between the output of the filter and the unknown system was minimized [19]. The literature [20] has attempted to enhance the performance by changing the selection mode of the original structure. In this paper, a novel ABC-based positioning algorithm is proposed to determine the MS location in wireless communication systems. The migration operator of the biogeography-based optimization (BBO) approach with the ABC algorithm is proposed to solve poly-phase code design [21].

In most areas, users may face the restriction of poor hearability, and MS may not detect more than three BSs for location purposes in wireless communication systems. This paper applied an approach in which the MS’s coordinate needs to be improved when three BSs are available. Three TOA circles can be generated from the distance measurements. The distance’s relationship between the intersections of three circles with the estimated location can be defined as an objective function. The approach was accomplished by minimizing the nonlinear objective function under nonlinear geometrical constraints. ABC was employed here to search for the optimal MS location in the range of feasible intersections. The proposed algorithm can be applied not only to time-based technologies but also the circles generated from signal strength measurement. In the simulation results, the Taylor series algorithm (TSA) [22], linear lines of position algorithm (LLOP) [23], and range-scaling algorithm (RSA) [24] were used for wireless location estimation. The results of the proposed algorithm and other different algorithms were compared to each other. Lastly, the performance of the proposed algorithm based on ABC was compared to GA, including the positioning accuracy, efficiency, and convergence speed. The simulation results show that the proposed location algorithm can mitigate the NLOS ranging errors and improve the positioning performance.

This paper is organized as follows. In Section 2, we will introduce some related localization methods, including TSA, LLOP, RSA, and GA. Section 3 describes the detailed procedure of the ABC algorithm. Section 4 derives the MS location from the proposed algorithm based on ABC. Then, Section 5 discusses the performance of the mentioned methods according to numerical simulations. Eventually, the conclusion is presented in Section 6.

## 2. Related Works

### 2.1. Classical Localization Methods

In TSA, at least three BSs were detected for estimating the MS location because of the constraint of hearbility. By linearizing the TOA equations based on the Taylor series algorithm, only the first two terms were retained to obtain the matrix relations [22]. The LLOP algorithm resolved the MS location by utilizing the linear equations derived from subtracting any two original range nonlinear equations [23]. In the literature [24], RSA, which was a very typical location technique and could estimate the true range in NLOS environments, was proposed. Many developments of the positioning algorithms focus on mitigating NLOS error, and RSA was a method with excellent performance. To decrease the NLOS error, this positioning problem is designed as a constrained nonlinear optimization problem according to the geometry relationship of the cell layout.

### 2.2. Probabilistic Localization Methods

As the procedure of obtaining the target positions in the NLOS condition can be well approximated to solve a nonlinear system, several algorithms [25,26,27,28] have been developed to find the solutions with high accuracy. The scheme in [25] classified the area covered by the BS signals into LOS and NLOS zones by employing support vector classification and trigonometric approximation. In [26], the model of target locations was expressed as a probability distribution function. By processing the previous localization results with the algorithm known as two-step weighted least-squares, reduced NLOS error was achieved. The authors of [27,28] advanced the conventional particle filter (PF) algorithms to achieve enhanced computational efficiency and reduced complexity when solving the nonlinear localization systems.

### 2.3. Localization Methods Based on Deep Learning

With the rapid development of deep learning, the performances of target positioning can be further improved by employing iterating calculations to reduce the NLOS errors [29,30,31,32]. In [29], hierarchical voting, known as the policy-based algorithm, was conducted before the measured signals enter the conventional filters. The authors of [30] processed the database of AOA signals with deep convolutional neural (DCN) networks. The performance of location prediction was improved by optimizing the coefficient weights in DCN. The algorithm based on the deep neural network in [31] was capable of reaching convergence fast under noisy and varying environments. A gradient-related operation named proximal policy optimization (PPO) was executed on AOA measurement to correct the NLOS variances in the positioning results [32].

The author of this paper proposed a genetic algorithm (GA)-based method to estimate the MS location [33]. GA was utilized to determine the MS location in this positioning optimization problem. First, the coordinates were transformed to the binary chromosome by an encoding scheme. A solution to a problem was represented as a chromosome in the population. Then, the fitness of each chromosome was calculated in the reproduction scheme. We chose the chromosomes with a better value of the objective function, to generate the next generation. Crossover and mutation schemes could promote the performance of an individual’s multiplicity by modifying the chromosome sequence. The chromosomes approached the optimal location gradually during the iterations. The estimated MS location was obtained from decoding the best chromosome until the convergence conditions meet.

### 2.4. Three-Dimensional (3-D) Target Localization Methods

The authors of [34] assisted an accurate indoor localization method based on the time- and angle-domains data of multipath transmissions with an existing 3-D map of the indoor conditions. The paper [35] employed the modified least-squares model to advance the accuracy of measuring the target coordinates on the *Z*-axis in 3-D environments. Furthermore, the selection of an optimal set of base stations according to their space distributions was considered as well. To construct a model of depicting the NLOS transmissions, the method of [36] was proposed to identify the obstructions in 3-D surroundings.

## 3. Artificial Bee Colony Algorithm

In this section, the detailed introduction of the ABC algorithm will be described. Bees distribute the foraging information to members in other colonies, and such behaviors can be observed according to the behavior of the real bees [37]. In the optimization problem, the value of the food source always corresponds to the objective function value. That is to say, the value of the food source increases and decreases with the objective function value and the best food source can be derived from the hive in the end of the algorithm. Different kinds of bees search for the best food source by using several mechanisms. The parameter of colony size would be set before implementing the ABC algorithm. Colony size is the amount of all bees. The amount of all bees is divided into two halves, one half is applied by employed bees, and another half is used by onlookers. There are three groups of bees in the ABC algorithm: employed bees, onlookers, and scouts. The employed bees are responsible for searching for the initial food sources and sharing their information with other bees. The onlookers decide the searching range based on the information sent from the employed bees. Scouts search the whole environment randomly. The ABC algorithm consists of these main components and makes use of these components to find the best solution.

### 3.1. Initialize Solutions

At the beginning of the ABC algorithm, the colony size would be defined. The initial solutions are randomly generated in the searching range. The number of food sources is equal to the half-colony size. The random solutions are defined as:

(1) $$x_{i,j}=x_{\min,j}+rand(0,1)(x_{\max,j}-x_{\min,j}),$$

where $i=1,2,\ldots ,N_{p}$, $N_{p}$ and j are food sources and the dimension, respectively. The number of dimensions represents the number of optimization parameters. Each initial solution is equal to a position of the food source. The food sources will attract bees to make honey. Then, each objective function value at the food source should be calculated and memorized.

### 3.2. Employed Bee Phase

At the beginning of the employed bee phase, each employed bee is associated with a food source. Therefore, the amount of food sources is equal to the number of employed bees. They send the data of the distances and directions of food sources to others in the nest. The employed bee produces a new modification from initial food sources, and employs greedy selection between the old one and the new one. There are five steps to model the employed bee phases:

(i) Let $x_{kj}$ be the neighbor food source, for *k* = rand (1, *Np*).
(ii) Find a new food source near the current food source; the new food source can be derived from:
  (2) $$v_{ij}=x_{ij}+\varphi_{ij}(x_{ij}-x_{kj}),$$
  where $\varphi_{ij}$ = rand (-1, 1), $i=1,2,\ldots ,N_{p}$, and j is the dimension.
(iii) Calculate the objective function value of $v_{ij}$.
(iv) Compare the objective function values of $x_{ij}$ and $v_{ij}$.
(v) Bees substitute a new memory of the food source $v_{ij}$, which has a better objective function value, into the old one of $v_{ij}$. On the other hand, $v_{ij}$ is reserved if it has a worse value.

After each employed bee completes its task of searching, a waggle dance is performed to show the onlookers the positions of the food sources.

### 3.3. Onlookers’ Phase

During the onlookers’ phase, they are standing by until employed bees finish the food-searching procedures. They receive information of the food sources from employed bees. However, different from the employed bees, onlookers select the food sources to develop according to the nectar amount. On the other hand, the food source with a better objective function value has a higher probability to select for searching near it. Then, the following procedures are similar to worker bees. Each onlooker provides a new food source near the previous one and applies the greedy selection between them. The detailed steps are described as follows:

(i) First, define the $fit_{i}$ function for $i=1,2,\ldots ,N_{p}$:
  (3) $$fit_{i}=\frac{1}{\left(1+f(x_{i})\right)}.$$
(ii) From (i), one can derive the probability value:
  (4) $$\begin{array}{cc} probability_{i}= & \frac{fit_{i}}{\sum_{i=1}^{Np}fit_{i}}.\\ x_{i,j}= & x_{\min,j}+rand(0,1)(x_{\max,j}-x_{\min,j}) \end{array}$$
(iii) The onlooker chooses the food source to search depending on the *probability*. Let $v_{kj}$ be the neighbor food source, for *k* = rand (1, *Np*).
(iv) Express the relation between the selected and a new food source by the following equation:
  (5) $$v_{ij}{}^{\prime }=x_{ij}+\varphi_{ij}(v_{ij}-v_{kj}),$$
  where $\varphi_{ij}$ = rand (−1, 1),$\;i=1,2,\ldots ,N_{p}$ and j is the dimension.
(v) Calculate the objective function value of $v_{ij}\prime$.
(vi) Compare it to the objective function value of $v_{ij}$ with $v_{ij}\prime$.
(vii) Substitute a new memory of the food sources $v_{ij}$, which has a better objective function value, into the old one of $x_{ij}$. On the other hand, $v_{ij}$ is reserved if it has a worse value.

After this selection, each onlooker bee searches for a new position based on the probability value.

The onlookers guarantee to provide better food sources than the previous food sources provided by employed bees.

### 3.4. Scouts Phase

In ABC, scouts are in charge of searching the whole range surrounding the nest if the abandoned food source exists. In the scouts’ phase, a parameter called limit would be defined. The value of limit, which is used to determine the abandoned case, presents the upper bound of the searching number without any improvement. The memory in the ABC algorithm records the number of times that the food source does not improve. When the frequency exceeds limit, the food source should be abandoned and transformed to a scout. A scout randomly searches for a new food source in the whole solution space. The food source supported by the scout replaces the abandoned food source.

(i) Determine whether the searching number without improvement exceeds the limit value.
(ii) If the abandon case exists, the food sources are changed to the scouts.
(iii) Send scouts to randomly find new food sources in the whole searching range by:
  (6) $$s_{i,j}=s_{\min,j}+rand(0,1)(x_{\max,j}-x_{\min,j}),$$
  where $i=1,2,\ldots ,N_{p}$ and j is the dimension.
(iv) The food sources derived from scouts are substituted for abandoned food sources directly.

In each cycle, the ABC algorithm creates a new population in the searching range. Then, the employed bee phase, onlookers’ phase, and scouts’ phase are implemented serially. For the final cycle, the ABC algorithm memorizes the best solution and determines whether the formulated requirements are satisfied. If the convergence condition is satisfied, the loop will be terminated and the best food source will be outputted as the optimal solution. Otherwise, the bees go back to the employed bee phase. The cycles are iterated continuously until the condition is satisfied. Generally, the best solution can be derived from the optimal procedure of the ABC algorithm during the iterations.

## 4. The Proposed ABC-Based Location Estimation

Researchers have proposed various positioning methods to estimate the MS location more accurately, such as TSA, LLOP, and RSA. We also have provided the MS location estimation based on GA before [33]. The GA-based location method has a better performance than the RSA and other existing methods in our previous research. However, the general disadvantages of GA are the trapping in the local optimum and the lack of a mechanism to memorize the best individuals during the iterations [38]. Additionally, a poor local search ability, risk of a suboptimal solution, and delayed convergence are worrying problems also.

To overcome the main disadvantages above, instead of GA, the ABC algorithm is implemented on function minimization in this paper. The employed selection operation is one important difference between the GA and ABC algorithm. The selection operation of GA depends on the fitness performance. In the ABC algorithm, new solutions are produced with equal chance. The iteration of the ABC algorithm is according to the self-adjusting operation and memory mechanism. Compared to GA, the ABC algorithm can get out of a local minimum and attain the global optimum with relative computational simplicity for multivariable function optimization [37]. Another advantage of ABC is its simplicity. Parameters of GA are hard to set prior, but ABC is easy to implement and there are few parameters to adjust. The ABC algorithm is expected to provide the estimated MS location more accurately and efficiently, and to converge faster. In this study, a novel positioning method based on the ABC algorithm for MS location estimation is developed and the results are compared with the other existing methods.

In this section, the location model and objective function will be introduced first. Then, the proposed ABC-based location algorithm will be presented clearly. ABC is an optimum approach method that simulates the ability of bee colonies. ABC dynamically adjusts the value to fit a better solution based on the gathered environmental information. In this scheme, the ABC-based positioning algorithm is applied for optimal estimation. MS location estimation is accomplished by approaching a nonlinear optimization under nonlinear constraints. The object of optimization will be designed according to the question waiting to be solved. In this paper, the ABC algorithm is proposed to decrease the NLOS error and promote the positioning accuracy for wireless location systems.

**Figure 1:**
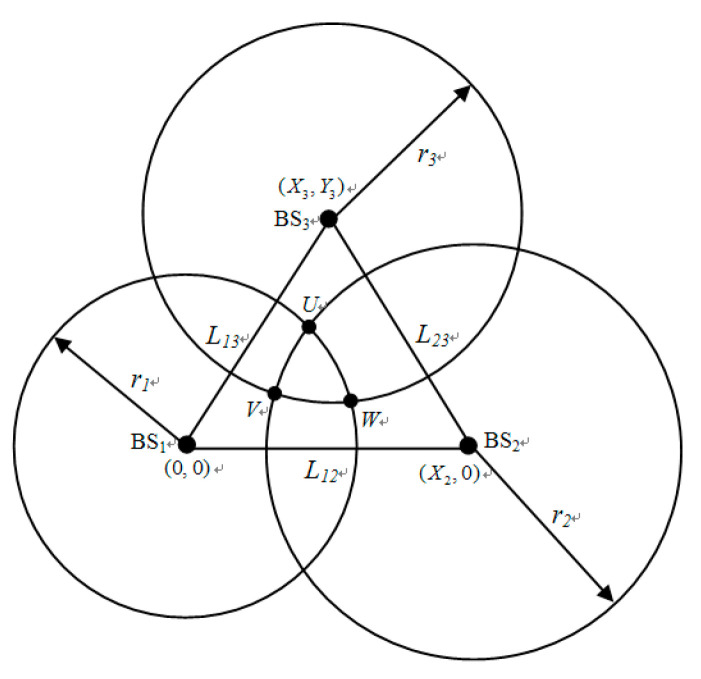
Geometric layout of the three circles.

The number of BSs is three due to the constraint on hearability. It is assumed that we have a transmitting MS, and TOA measurements are taken at each receiving BS. As shown in Figure 1, the coordinates for BS1, BS2, and BS3 are given by (0, 0), (*X*_2_, 0), and (*X*_3_, *Y*_3_), respectively. (*x*,*y*) is the MS coordinate waiting to be estimated. Then, the measured distance between the MS and the BS*_i_* is *r_i_*, *i* = 1, 2, 3. According to the geometric approach, each measured distance by a BS forms a circle, and the center of a circle is a BS. Multiple TOA measurements estimate MS location by the intersections of the circles. The following three equations indicate the circles for TOA measurement:

*Circle* 1: *x*^2^ + *y*^2^ = *r*_1_^2^, (7)

*Circle* 2: (*x − X*_2_)^2^ + *y*^2^ = *r*_2_^2^, (8)

*Circle* 3: (*x − X*_3_)^2^ + (*y-Y*_3_)^2^ = *r*_3_^2^. (9)

In an ideal case, the intersection of three circles would be a point. However, in a practical case, NLOS error is very common in our life. The accuracy of the estimated location degrades seriously because of the NLOS propagation environment. No matter what kind of ranging technique is used, the measured distance is greater than the true value. In the NLOS propagation environment, the measured distance is always increased due to the signal strength attenuation or propagation time delay. Therefore, the radius variation is always positive. Avoiding the interference generated from the NLOS error being too large is another problem, i.e., one circle is fully covered by another circle. Therefore, the measured radius would be adjusted so that any two circles can intersect at one point at least. If $r_{i}>L_{ij}+r_{j}$, we adjust the measured TOA value to $r_{i}=L_{ij}+r_{j}$ (i,j=1,2,3;i≠j), where $L_{ij}$ is the distance between BS*_i_* and BS*_j_*. It is also mentioned in [20] to ensure there is at least one intersection for any two TOA circles and to avoid the condition leads to the proposed location algorithm not being employed.

Because of the NLOS error, the circles partially overlap on each other, and the region is formed by the intersections. The true location of MS should be inside the overlap of three circles, which is surrounded by *U*, *V*, and *W*. *U*, *V*, and *W* are defined as the feasible intersections. Feasible intersections must meet the conditions of the following three inequalities:

(10) $$x^{2}+y^{2}\leq r_{1}{}^{2},$$

(11) $${\left(x-X_{2}\right)}^{2}+y^{2}\leq r_{2}{}^{2},$$

(12) $${\left(x-X_{3}\right)}^{2}+{\left(y-Y_{3}\right)}^{2}\leq r_{3}{}^{2}.$$

The MS location has to satisfy all the above Equations (10)–(12), so that it can be estimated by using the feasible intersections of three circles. The nonlinear objective function was proposed in [27], which can be seen as a cost function in this paper. It is the summation of the Euclidian distances between the MS and the three points intersected by three TOA circles. By solving any two circle equations of (7)–(9), three circles have six intersections. Then, the points of feasible intersections *U*, *V*, and *W* can be percolated by the inequalities of (10)–(12). The coordinates of *U*, *V*, and *W* are represented as (*U_x_*,*U*_y_), (*V_x_*,*V*_y_), and (*W_x_*,*W*_y_), respectively. Then, the cost function of the proposed ABC algorithm is as follows:

(13) $$f(x,y)=\sqrt{{\left(x-U_{x}\right)}^{2}+{\left(y-U_{y}\right)}^{2}+\;{\left(x-V_{x}\right)}^{2}+{\left(y-V_{y}\right)}^{2}+{\left(x-W_{x}\right)}^{2}+{\left(y-W_{y}\right)}^{2}}.$$

So, the considered positioning problem is reformulated to an optimizing problem. In the best situation, the feasible intersections of *U*, *V*, and *W* overlap with the minimization of the objective function value. The objective function value is equal to zero at this moment. By minimizing this objective function, the NLOS error can be decreased effectively. The considered optimizing problem can be described as minimizing the cost function with nonlinear constraints. The general constrained optimization problem is to find the MS location (*x*, *y*), so as to:

(14) minimize f(x,y) .

With the constraints represented as:

(15) $$\begin{array}{l} \min\{U_{x},V_{x},W_{x}\}\leq x\leq \max\{U_{x},V_{x},W_{x}\}\\ \min\{U_{y},V_{y},W_{y}\}\leq y\leq \max\{U_{y},V_{y},W_{y}\} \end{array}$$

**Figure 2:**
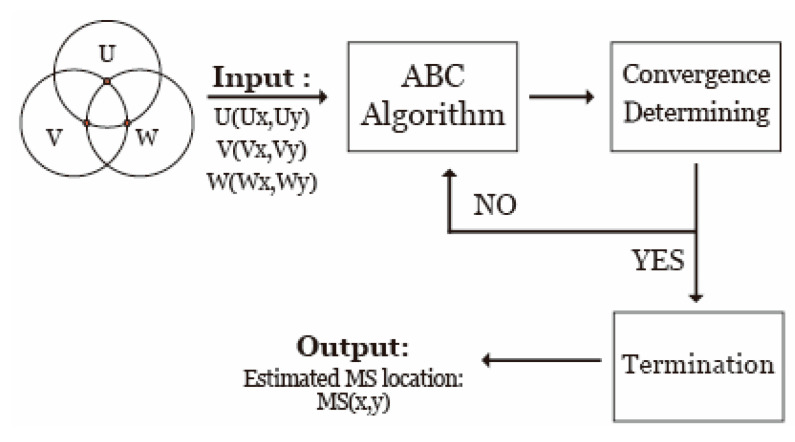
Main procedures of the proposed artificial bee colony (ABC)-based algorithm.

In this scheme, he ABC algorithm is applied to solve the constrained optimization problem. Each bee represents a horizontal coordinate and each food source is considered as a possible solution. In the ABC algorithm, two control parameters should be set *prior*, including the colony size and the *limit* value. Because of the differences in the searching range of the *x* and *y* coordinates, the coordinates of *x* and *y* cannot be regarded as a parameter with two dimensions. The ABC-based positioning algorithm is completed by operating on two parameters for the *x* and *y* coordinates to search for the best solution separately and simultaneously. However, the two parameters for the *x* and *y* coordinates are used together to calculate and compare the objective function value. In this step, the searching range is shown as Equation (15). If the ABC algorithm is regarded as a block diagram, the proposed positioning method can be simply presented as Figure 2. After the feasible intersections of *U*, *V*, and *W* are obtained, the proposed positioning method utilizes the ABC algorithm to estimate the MS location.

Honey bees use several mechanisms like the waggle dance to locate food sources and search for new ones optimally. By the behaviors of a honey bee colony, the richest food sources can be obtained in the shortest possible time. The position of the richest food source is the estimated MS location with the highest opportunity approaching the real MS location. The convergence qualification is defined as the solution that keeps the minimization of the objective function value during three cycles continuously. During iterations, the best solution can be found within the area bounded by feasible intersections of three circles and satisfies the convergence qualification with the lowest objective function value.

## 5. Simulation Results

**Figure 3:**
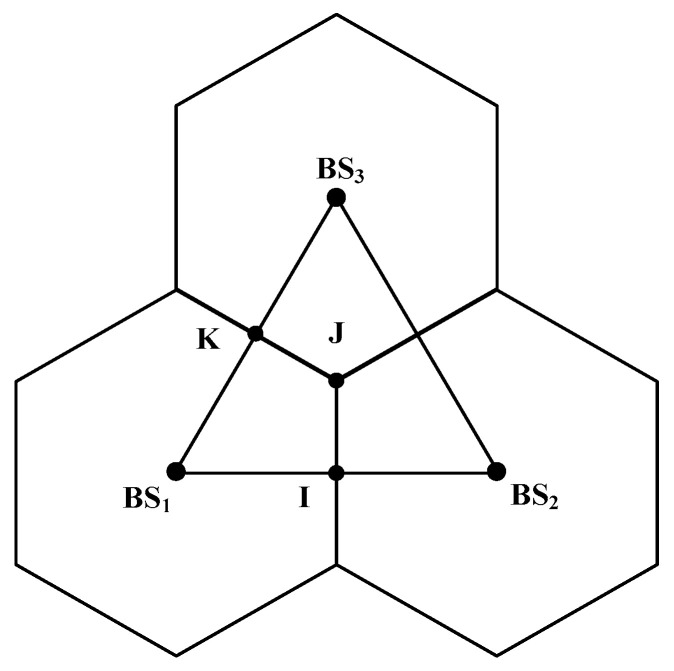
Cell layout showing the relationship between the base stations (BSs) and the inter-BS distances.

To examine the performance of the proposed location algorithm in the NLOS environment, computer simulations should be implemented. The performance of the proposed location algorithm was compared with other algorithms, such as TSA, LLOP, RSA, and GA. In this paper, the three BSs coordinates were set to BS1: (0, 0), BS2: (1732 m, 0), and BS3: (866 m, 1500 m). For each test, the random real MS location was chosen with a uniform distribution in the range surrounded by the points BS_1_, *I*, *J*, and *K* as shown in Figure 3. In total, 10,000 tests were performed independently and all numerical quantities are presented in meters.

**Figure 4:**
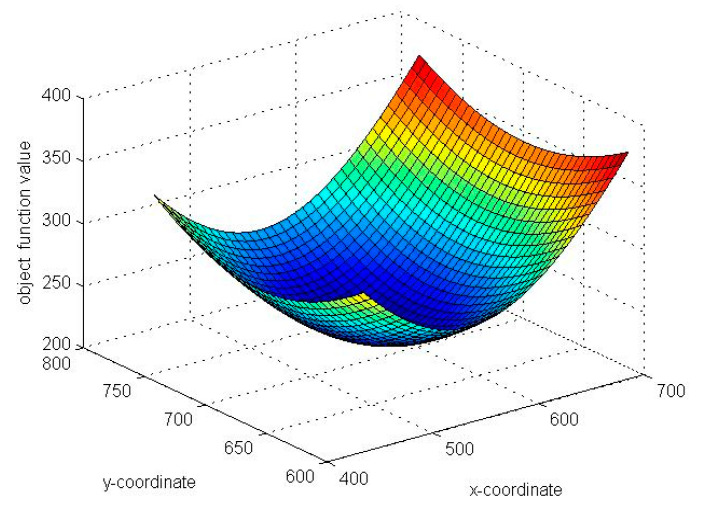
The surface plot of the objective function.

Before the ABC algorithm was implemented, some control parameters were defined. The control parameters were set to colony size = 100, and *Limit* = 10. Another control parameter is maxcycles, defined as the max cycles of iteration. The control parameter of maxcycles does not need to be discussed in this research. Rather than apply the fixed parameter, we utilized the convergence mechanism to decide the moment for stopping the iterations. Many test functions were used to observe the performance in [39]. The objective function we used in this paper is just like a Griewank function. The corresponding global optimum solution is the global minimum value, which is the bottom of the surface for this function, as shown in Figure 4. It can be observed that the food source in the ABC algorithm is strongly multimodal when the objective function value decreases with dimensionality. The bees search in the limited range until the optimal solution can be found. The optimal solution is the prediction of the MS location.

**Figure 5:**
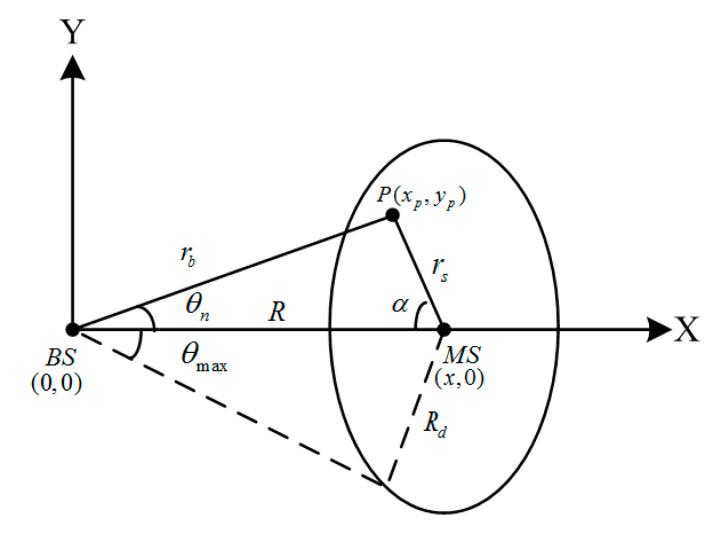
Geometry of circular disk of scatters model (CDSM).

The NLOS error is the main reason that causes location accuracy with a bad performance. NLOS effects were taken into account for the analysis of simulations in this paper. Two typical error models were applied, one is the circular disk of scatters model (CDSM) [40] and another one is a uniformly distributed noise model [24]. CDSM is the first introduced error model, which simulates the NLOS propagation environment. It assumes that the path of signal transmission between MS and BSs is not a straight path. One scatter spreading around MS in the circular disk is considered to obstruct the signal transmission. The signal is changed to go through a single reflection on the points of scatter. Therefore, the measured distance is reformulated by calculating the sum of two distances, including the distance between BSs and the scatters, and the distances between MS to the scatters. The measured distance would be increased according to the triangle theorem shown as Figure 5. *P* presents the scatter point. The real travel path is between MS and BS, but the measured ranges are the sum of *r_b_* and *r_s_* after the signal is interfered with by the scatter point. The AOA measurement is also affected in the CDSM error model. *θ_n_* is the AOA measurement error. The scatter points in the circular disk are considered as the lower range error with a higher probability to appear. The maximum error value is when the scatter point is on the circumference. Thus, range error depends on the scatter radius; a bigger scatter radius with a larger measured error.

**Figure 6:**
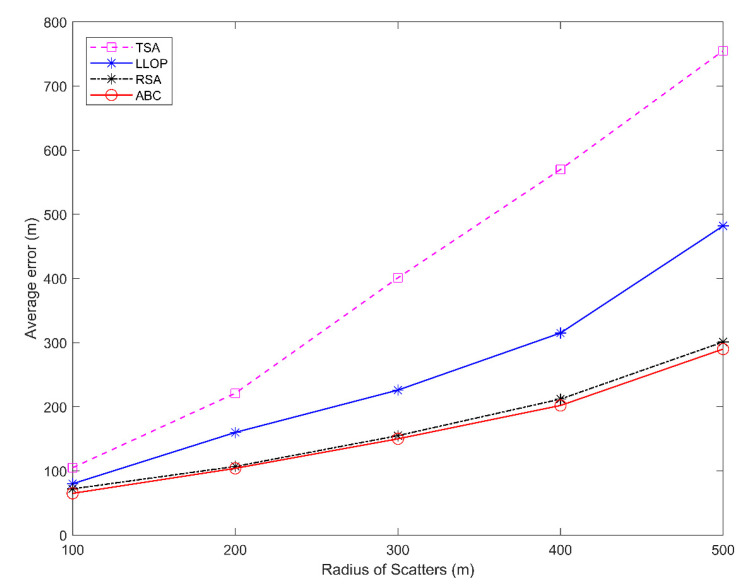
Average location error versus the different radius of scatters.

Figure 6 shows the average location error of the proposed ABC-based location algorithm compared with other existing methods in the CDSM error model. All methods increased the average error, with the longer radius of scatter. However, the proposed ABC-based algorithm has a lower average location error than TSA, LLOP, and RSA. The curve of the method we proposed has a relatively smooth curve. Compared with TSA, LLOP, and RSA, the simulation results show that the proposed algorithm has the best performance on the location accuracy and supports the estimation of MS location with the best accuracy.

**Figure 7:**
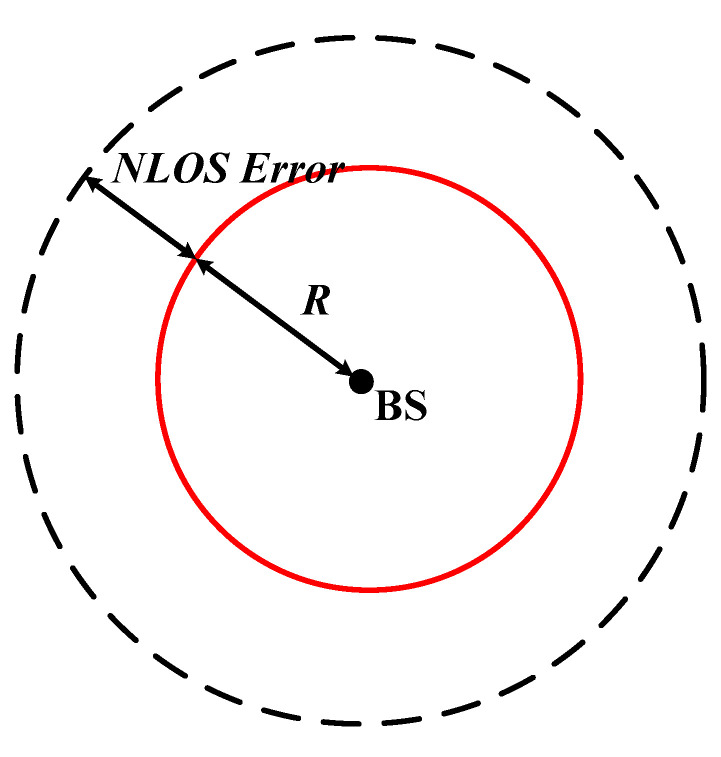
Example of the uniformly distributed noise model.

**Figure 8:**
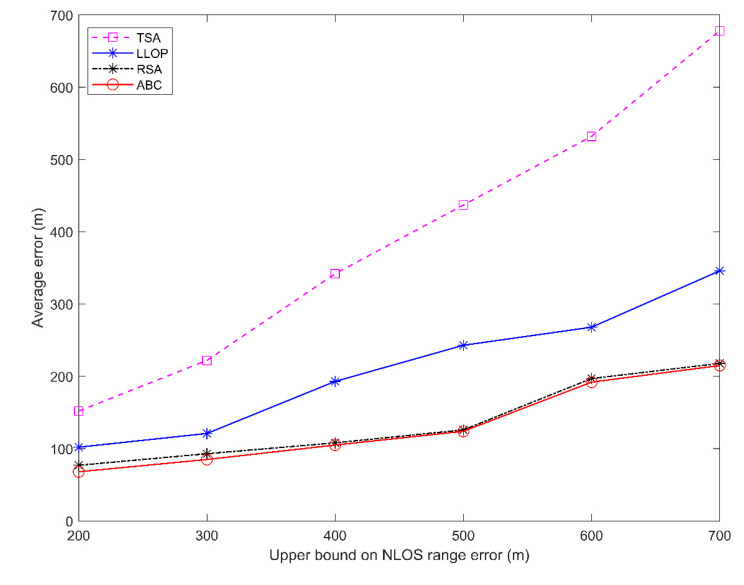
Average location error versus the upper bound of non-line-of-sight (NLOS) errors.

The second common NLOS propagation model is the uniformly distributed noise model [27]. In this NLOS error model, the measured distance is the true distance, which adds an error value. The NLOS error value is distributed uniformly in the range (0, *U_i_*), where 0 and *U_i_* are the lower bound and the upper bound of the error for each BS. An example of a uniformly distributed noise model is shown in Figure 7. The measured distance is the sum of the real distance *R* and the NLOS error with a uniform distribution for each BS. In Figure 8, the simulation shows the performances of the proposed algorithm and other methods with the NLOS error of various upper bound values. Obviously, the proposed algorithm has the lowest average location error in the uniformly distributed noise model.

In a previous study, we proposed the positioning algorithm combined with GA [33]. A positioning method based on the ABC algorithm is used to precisely locate the MS as well as accelerate the convergence time, which is a bottleneck of the one based on GA. Therefore, the comparison of ABC and GA is an important issue. The error effect of CDSM and the uniformly distributed noise model on the average location error was compared with GA and ABC, as shown in Table 1 and Table 2, respectively. Both the GA and ABC algorithms provide a more accurate estimation of the MS location. It can be observed that the ABC-based algorithm has a better performance than GA-based in the two NLOS error models.

**Figure 9:**
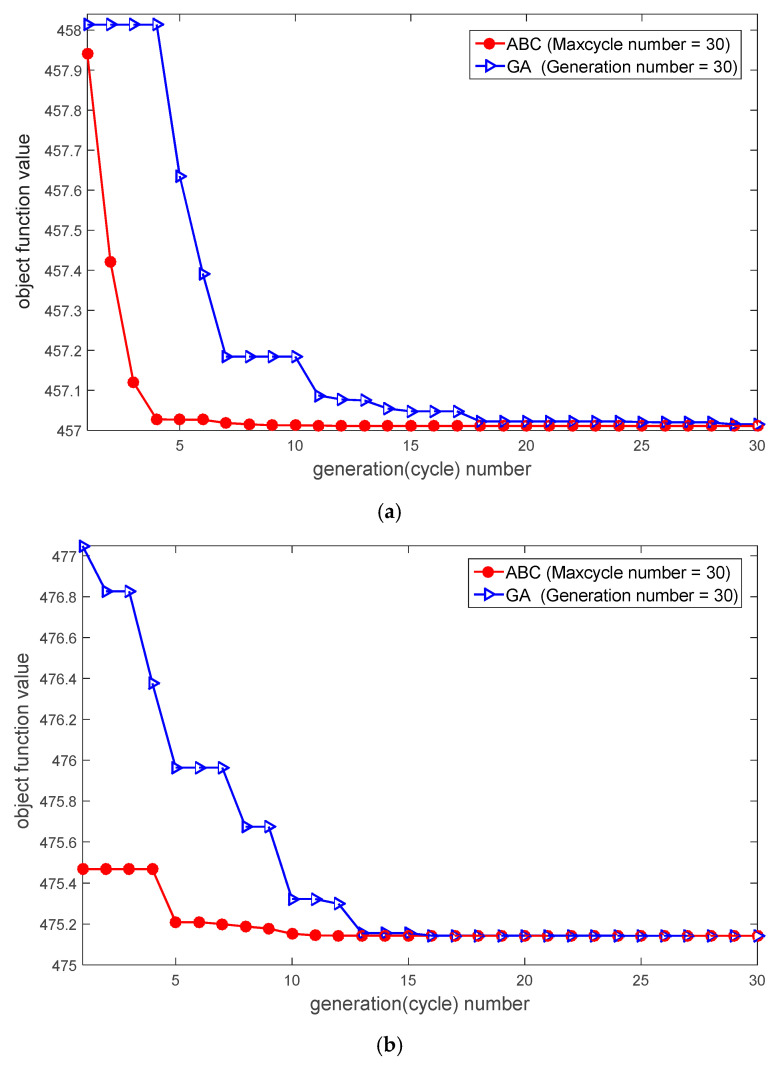

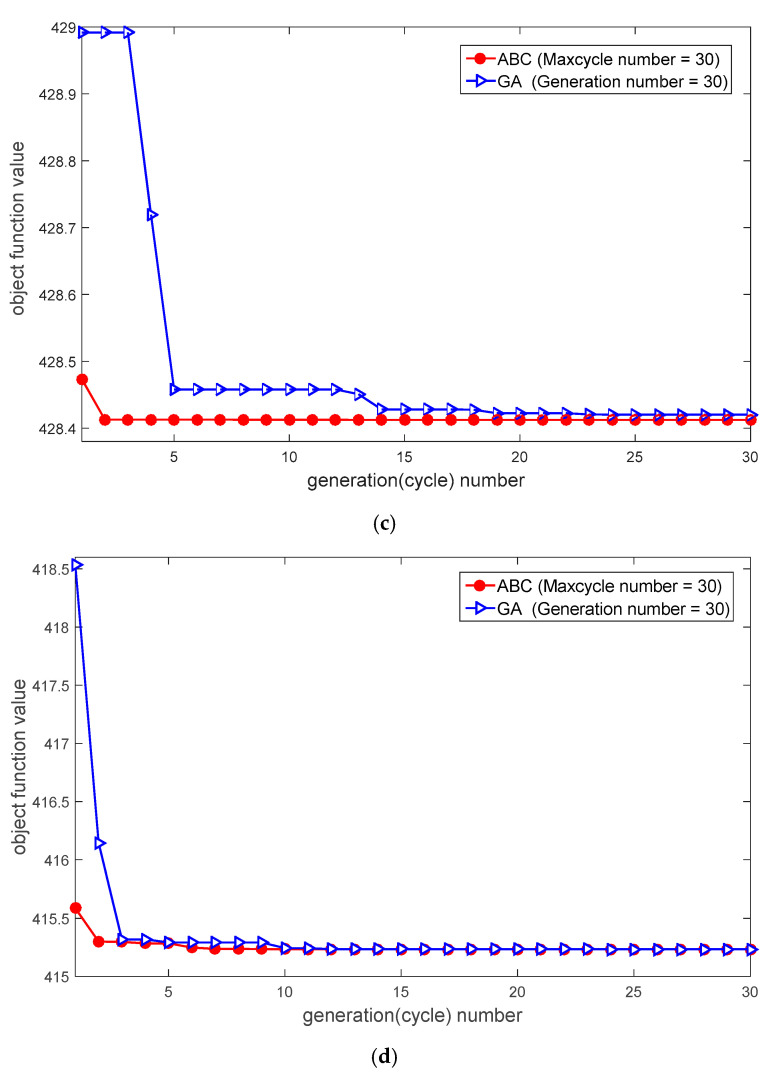
Convergence graph of the best objective function value in different error models: (**a**) The radius of scatters of 200 m on the CDSM error model (**b**) The radius of scatters of 400 m on the CDSM error model (**c**) The upper bound of 300 m of NLOS range error (**d**) The upper bound of 500 m of NLOS range error.

Another important ability is the efficiency of positioning, as how to provide the most accurate MS location in the shortest time is a critical and practical issue. Consequently, in order to provide the MS location estimation efficiently, the speed of convergence is a significant indicator. The algorithm with faster convergence can reduce the consumption time and computational complexity for positioning. As a result, the proposed algorithm not only reduces the time for positioning but also saves redundant hardware resources. Figure 9 demonstrates the proposed ABS-based algorithm has a quick convergence in various error models. In each error model, the proposed algorithm has the fast converging ability to obtain the best solution by the swarm intelligence of bees. The average convergence cycles/generations and the time taken for the procedure are recorded in Table 3. The results show that not only are the iteration cycles/generations of the ABC algorithm less than GA but also the ABC algorithm needs less time to implement the optimization problem when the number of loops is fixed. Compared to GA, the ABC algorithm has a relatively simple and fast-converging iterative procedure. It can be observed from our simulation results that the proposed ABC-based location algorithm can improve the location accuracy and speed up the positioning execution time by its excellent optimization ability.

## 6. Conclusions

In this paper, a novel ABC-based location algorithm was presented to estimate the MS location in the NLOS environment. The objective function of the proposed location algorithm is according to the geometrical relationships between MS and three BSs. It can be observed that the NLOS interference can be ameliorated when the sum of the distance from MS to three feasible intersections is minimized. Therefore, the problem of MS location estimation can be regarded as a constrained optimization problem. The ABC algorithm was applied to search for the MS location in this constrained optimization problem. The ABC algorithm is a swarm intelligence algorithm, based on the behavior observed from the food source searching procedure of the honey bee. Three kinds of bees search for the best food source in the searching range, which overlap the three circles. During cycles, the value of the objective function is decreased gradually. Lastly, the optimum solution, which equals the estimated MS location, could be obtained by the ABC algorithm without any NLOS error prior information. The simulation results of the location error show that the positioning accuracy of the proposed ABC-based location algorithm is better than LLOP, TSA, RSA, and GA. Compared to GA, the ABC-based algorithm is more accurate and efficient, converges faster, and is capable of handling complex optimization problems. Another important advantage is that the ABC algorithm is relatively simpler compared to other well-known metaheuristic algorithms like GA, as only two parameters (colony size and limit) are needed in the ABC algorithm. In this paper, the proposed ABC-based location algorithm provides the estimated MS location not only with best location accuracy but also the shortest execution time.

## Figures and Tables

**Table 1:** Average location errors versus the radius of scatters.

Radius of scatter (m\m)	100	200	300	400	500
GA-based algorithm	64.32	121.71	174.5	232.71	292.93
ABC-based algorithm	62.84	117.37	173.91	231.56	290.59

**Table 2:** Average location errors versus the upper bound of the NLOS errors.

Upper bound (m\m)	200	300	400	500	600	700
GA-based algorithm	65.59	94.97	123.58	154.82	186.58	218.2
ABC-based algorithm	65.16	93.37	122.74	153.49	185.14	218

**Table 3:** Average convergence cycles/generations and the location execution time.

Method	ABC-Based Algorithm	GA-Based Algorithm
Convergence	12.98 (cycles)	26.62 (generations)
Time (for 30 cycles/generations)	4.51 (s)	6.93 (s)
Time (convergence)	2.40 (s)	6.14 (s)
